# Arginine68 is an essential residue for the C-terminal cleavage of human Atg8 family proteins

**DOI:** 10.1186/1471-2121-14-27

**Published:** 2013-05-30

**Authors:** Chao Liu, Haijie Ma, Jiaxue Wu, Qiang Huang, Jun O Liu, Long Yu

**Affiliations:** 1 State Key Laboratory of Genetic Engineering; Institute of Genetics; School of Life Sciences, Fudan University, Shanghai 200433, China; 2 Departments of Pharmacology and Molecular Sciences and Oncology, Johns Hopkins University School of Medicine, Baltimore, MD 21205, USA; 3 Institute of Biomedical Sciences, Fudan University, Shanghai 200433, China

**Keywords:** Atg8, MAP1LC3B, Autophagy, Alternative splicing

## Abstract

**Background:**

Autophagy is a conserved cellular process that degrades and recycles cytoplasmic components via a lysosomal pathway. The phosphatidylethanolamine (PE)-conjugation of the Atg8 protein plays an important role in the yeast autophagy process. In humans, six Atg8 homologs, including MAP1LC3A, MAP1LC3B, MAP1LC3C (refer to LC3A, LC3B, and LC3C hereafter), GABARAP, GABARAPL1, and GABARAPL2 have been reported. All of them can be conjugated to PE through a ubiquitin-like conjugation system, and be located to autophagosomes.

**Results:**

In this study, we found 3 new alternative splicing isoforms in LC3B, GABARAP, and GABARAPL1, (designated as LC3B-a, GABARAP-a and GABARAPL1-a, respectively). None of them can go through the PE-conjugation process and be located to autophagosomes. Interestingly, compared with LC3B, LC3B-a has a single amino acid (Arg68) deletion due to the NAGNAG alternative splicing in intron 3. Through structural simulations, we found that the C-terminal tail of LC3B-a is less mobile than that of LC3B, thus affecting its C-terminal cleavage by human ATG4 family proteins. Furthermore, we found that Arg68 is an essential residue facilitating the interaction between human Atg8 family proteins and ATG4B by forming a salt bridge with Asp171 of ATG4B. Depletion of this salt bridge reduces autophagosomes formation and autophagic flux under both normal and nutrition starvation conditions.

**Conclusions:**

These results suggest Arg68 is an essential residue for the C-terminal cleavage of Atg8 family proteins during the autophagy process.

## Background

Macroautophagy (hereafter referred to as autophagy) is an evolutionarily conserved cellular process which degrades and recycles cytoplasmic components through the *de novo* synthesized membrane system and lysosome [1,2]. The activity of autophagy is important for cellular homeostasis, cell survival, development, and aging [3]. Deregulated autophagy results in many physiological defects, including liver injury, muscular disorder, neurodegeneration, pathogen infections and cancer [4-9].

In response to stress conditions, such as starvation, autophagy can be up-regulated. Cytoplasmic components are sequestered into an expanding phagophore, which finally encloses to form double membrane vesicles, named autophagosomes. Autophagosomes are transported and fused with lysosomes, then the inner membrane and cargo can be digested by multiple enzymes existing in lysosomes. The resulting small molecules are finally transported out of the lysosomes for recycling [1].

In the past 16 years, molecular genetic studies in yeast have identified at least 32 autophagy-related genes (ATG) functioning in different steps of autophagy [10,11]. Among them, the ubiquitin-like protein Atg8 is the only protein that could bind to all autophagic membrane structures, thus it is commonly used as a marker to trace autophagosomes [12]. Before binding with autophagic membrane structures, newly synthesized Atg8 protein (pro-Atg8) is processed by a cysteine protease, Atg4. Thus the C-terminus is cleaved and the conserved glycine residue is exposed, forming Atg8-I molecules [13]. Then Atg8-I protein is covalently conjugated with PE through a ubiquitin-like conjugation system, including E1-like enzyme Atg7 [14,15], E2-like enzyme Atg3 [16], and E3-like enzyme Atg12-Atg5-Atg16 complex [17]. The PE-modified Atg8 (Atg8-II protein) is relocated to the autophagic membrane structures and mediates the membrane tethering, expanding, and cargo recognition [18-21].

In humans, Atg8 homologs comprise of six members: LC3A, LC3B, LC3C [22-25], GABARAP [24,26], GABARAPL1/ATG8L [27] and GABARAPL2/GATE-16 [24,28]. The conserved post-translational modification process on these Atg8 family proteins is essential for their biological function during the autophagy process [23,24,29-31]. To fully explore the family members of human Atg8 homologs, we analysed their alternative splicing patterns and found new isoforms for LC3B, GABARAP, and GABARAPL1, designated as LC3B-a, GABARAP-a and GABARAPL1-a respectively. Interestingly, comparing to LC3B, LC3B-a lacks only one amino acid (Arg68) due to the NAGNAG alternative splicing [32], and has severely impaired C-terminal cleavage efficiency by ATG4B, one of the major members of the human ATG4 family proteins [29]. Furthermore, we found that Arg68 was also an essential residue for the interaction between Atg8 homologs and Atg4B in human. Mutation of Arg68 in human Atg8 family proteins would severely decrease their C-terminal cleavage efficiency, thus affecting their autophagic localization and autophagic flux. Taken together, we report the identification and characterization of an essential residue for the autophagic activity of human Atg8 family proteins, which provides potential targets to regulate the autophagy activity in human diseases.

## Results

### Identification of new isoforms in human Atg8 family proteins

**Figure 1:**
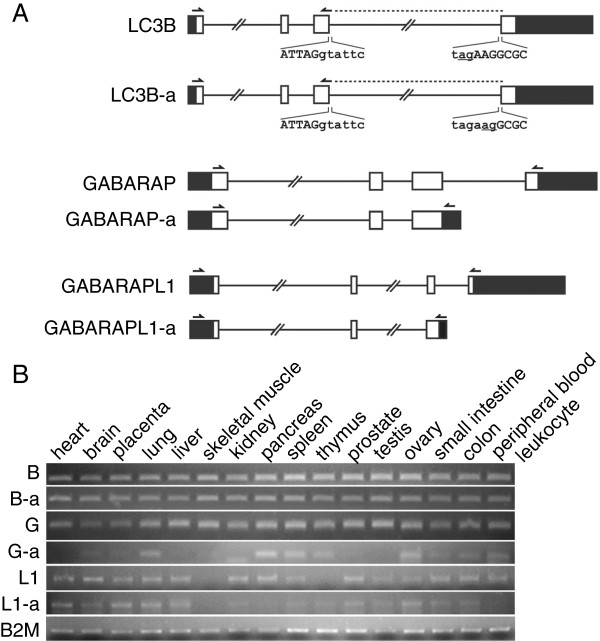
**Alternative splicing patterns and expressions of human Atg8 homologs. A**. Schematic diagram of the alternative splicing patterns of *LC3B*, *GABARAP* and *GABARAPL1* mRNA. White and black boxes represent the protein-coding or non-coding region, respectively. Straight lines represent introns. Nucleotide sequences at the intron 3 splicing sites are shown. Splicing acceptors are underlined. Arrows show the positions of the corresponding allele specific primers. **B**. Expression of each transcript in multiple human tissues. Semi-quantitative PCR for each transcript was performed on human multiple-tissue cDNA panels. The expression of housekeeping gene B2M was analysed as loading control. B, LC3B; B-a, LC3B-a; G, GABARAP; G-a, GABARAP-a; L1, GABARAPL1; L1-a, GABARAPL1-a.

Alternative mRNA splicing generates a diverse range of mature RNAs, and potentially expands the cellular protein repertoire [33]. Thus, one gene can be translated to multiple proteins with distinct biological functions. To fully explore the members in human Atg8 family proteins, we searched their alternative splicing patterns from the Alternative Splicing and Transcript Diversity (ASTD) database which has been integrated in Ensembl (http://www.ensembl.org) [34]. As shown in Figure 1A, we found new mRNA transcripts in *LC3B*, *GABARAP*, and *GABARAPL1*. We named their alternative transcripts as *LC3B-a*, *GABARAP-a*, and *GABARAPL1-a*, respectively [GenBank: JN663879, JN663880, and JN663881]. Specifically, the isoform of GABARAP-a or GABARAPL1-a adopt different 3’ ends, resulting the missing of the conserved glycine necessary for ATG4 cleavage and down-stream processing [35]. LC3B has two tandem 3’ splice acceptor sites (TAGAAG) in intron 3 (Figure 1A). Using the distal splice site (AAG) would remove AAG as part of the exon 4, thus resulting in the loss of Arg68 after translation.

To verify the existence of these transcripts, we cloned these transcripts from a HeLa cDNA library. The sequencing result for each transcript is shown in Additional file 1: Figure S1. We also amplified each transcript by allele-specific PCR from multiple human tissue cDNA libraries. Primer specificity for LC3B and LC3B-a were tested by using LC3B and LC3B-a plasmids as templates. As shown in Additional file 1: Figure S2, the primers we designed can distinguish both transcripts specifically. As shown in Figure 1B, all three newly identified transcripts exist in human tissues. While *LC3B*, *LC3B-a*, and *GABARAP* express ubiquitously in human tissues, *GABARAP-a* and *GABARAPL1-a* have tissue specificities. Furthermore, the expressed sequence tags (EST) supporting the existence of each transcript were shown in Additional file 1: Table S1.

### Three new isoforms could neither undergo post-translational modification nor be localized to autophagosomes

**Figure 2:**
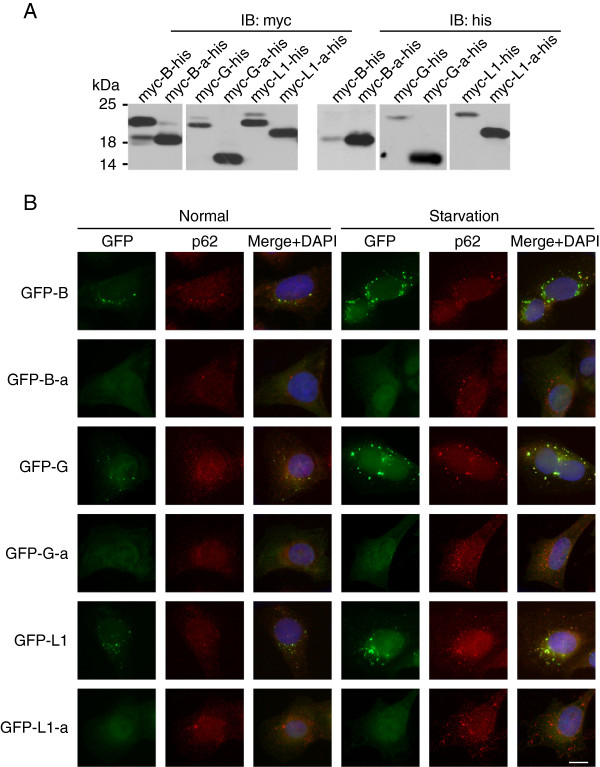
**The post-translational modification pattern and cellular localization of new isoforms. A**.The post-translational modification of isoforms in LC3B, GABARAP, and GABARAPL1. 24 hours after transfection, cell lysates of HeLa cells were subjected to SDS-PAGE followed by western blotting with anti-myc and anti-his antibodies. Molecular weights (kDa) is shown on the left. **B**. GFP tagged LC3B, LC3B-a, GABARAP, GABARAP-a, GABARAPL1, or GABARAPL1-a were stably expressed in HeLa cells. After treated with or without starvation for 2 hours, cells were subjected to immunostaining with p62 protein as autophagosome marker. B, LC3B; B-a, LC3B-a; G, GABARAP; G-a, GABARAP-a; L1, GABARAPL1; L1-a, GABARAPL1-a. Bar: 20 μm.

The removal of AAG as part of exon 4 results in the loss of Arg68 in the amino acid sequence of LC3B-a (Additional file 1: Figure S3). GABARAP-a and GABARAPL1-a lack the highly conserved β4 strand and Gly120 (Additional file 1: Figure S3), an indispensable amino acid for their C-terminal cleavage by Atg4 [35]. Thus, these two isoforms may not be processed by human Atg4 family proteins. To explore the post-translational modification patterns of these new isoforms, we first fused these proteins with myc-tag at the N termini and his-tag at the C-termini. Bands detected by anti-myc antibody show all forms of molecules after modification, including the pro form (non-cleaved form), the -I form (cleaved form) and the -II form (cleaved and PE conjugated form). Bands detected by anti-his antibody show only the pro form (non-cleaved form). As shown in Figure 2A, the C-terminal cleavages of LC3B-a, GABARAP-a, and GABARAPL1-a are all severely impaired. Large amounts of pro-form accumulate in the cell lysates.

Next, we fused GFP protein to the N-terminal of these proteins and examined their intracellular localization in HeLa cells under starvation conditions. As shown in Figure 2B, except for the three newly found members, all these proteins form puncta in the cytoplasm and co-localize to autophagosomal marker p62 [36]. In contrast, none of the new isoforms could be localized to autophagosomes but only ubiquitously disperses in the cytosol, even under starvation conditions.

These results show that the newly found isoforms could hardly be cleaved by human Atg4 family proteins, thus affecting their following modification process and autophagosomal localization.

### Conformational changes resulting from the loss of Arg68 inhibit the cleavage of LC3B-a by ATG4

The C-termini of GABARAP-a and GABARAPL1-a could not be cleaved by human Atg4 family proteins maybe due to the lack of the conserved Gly120, which is indispensable for C-terminal cleavage [35]. Interestingly, the only difference between the two isoforms of LC3B is the presence (in LC3B) or absence (in LC3B-a) of Arg68, suggesting that Arg68 may play an important role in the C-terminal cleavage of LC3B. To investigate the reason why the cleavage efficiency of LC3B-a from human ATG4 family proteins is severely affected, we first compared the interaction between ATG4B and these two isoforms. As shown in Additional file 1: Figure S4, both isoforms could be pulled down by ATG4B, suggesting that the interactions between ATG4B and these two isoforms are intact, and the low cleavage efficiency maybe due to other reasons.

**Figure 3:**
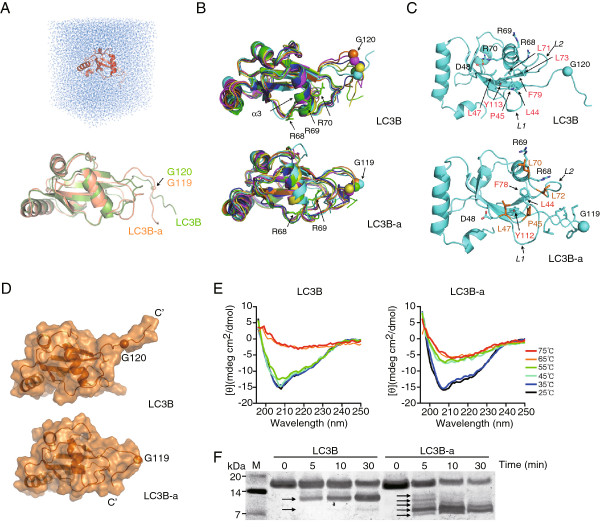
**LC3B-a has a different C-terminal tail conformation and less compact overall structure in solution. A**. The initial protein-water system for MD simulation (upper panel) and the homology-modeling structures of LC3B (green) and LC3B-a (orange) for MD simulations (lower panel). Only the backbones are illustrated by cartoon model. **B**. Superimposition of the simulation snapshots of LC3B or LC3B-a backbone at time t = 3 ns (green), 5ns (blue), 7 ns (yellow), 9 ns (magenta), 11 ns (orange), and 13 ns (cyan). **C**. The salt bridge between D48 and R70 and the hydrophobic cluster of L44, P45, L47, L71, L73, F79 and Y113 of LC3B are shown. No salt bridge is formed by D48 and R69 of LC3B-a and a weaker hydrophobic contact of L44, F78 and Y112 is presented in LC3B-a. The hydrophobic residues in the C-terminus of LC3B-a are shown as sticks. **D**. Space-filling model of the simulation snapshot of LC3B and LC3B-a at time t = 13 ns. The figures were generated using the program PyMOL[58]. C’: the C-terminus. **E**. Far-UV CD spectra of LC3B and LC3B-a in the temperature range of 25°C ~ 75°C. **F**. Time-dependent trypsin digestion patterns of LC3B and LC3B-a. Small fragments were shown by arrows. Molecular weights (kDa) were shown in the left lane “M”.

Then, we focused on the structure of human Atg8 family proteins. The structure of Atg8 [37], rat LC3 [38], GABARAP [39,40], GABARAPL2 [41] have been reported. They all contain 2 helices at the N-terminus and a conserved ubiquitin fold at the C-terminus. Arg68 is a conserved amino acid and located on the α3 helix of the ubiquitin fold (Additional file 1: Figure S3 and Figure 3B).As the α3 helix is the core of the ubiquitin fold, the deletion of Arg68 may affect the ubiquitin fold of LC3B-a. Taking advantage of the known crystal structures of three LC3B homologs: rat LC3 [38], GABARAP [40], and GABARAPL2 [41], we performed molecular modeling to assess the possible structural difference between LC3B and LC3B-a. The C-terminal fragments, including the conserved Gly120 in all three structures are disordered, and atomic coordinates of certain C-terminal residues are not available. Given the significant sequence conservation between LC3B and rat LC3, it was expected that the C-terminal tail of LC3B is also disordered in solution. We performed nanosecond molecular dynamics (MD) simulations to determine the motion states of the C-terminal segments of LC3B and LC3B-a in solution. We constructed the initial structures of both isoforms with the homology modeling method using the crystal structures of LC3B homologs as templates (see Methods). Two protein-water systems for MD simulations of LC3B and LC3B-a were then built (Figure 3A). Next, 13-ns MD simulations for each system were carried out. Since the protocols for the simulations of the two isoforms are the same, the resultant models of the two proteins can be compared to each other.

The modeling of LC3B showed that its C-terminal segment is quite flexible and disordered in solution, in agreement with those structures reported in the LC3B homologs [38,41] In the simulations, the C-terminal segment of about ten amino acids (Asn116 to Val125) were always projected into the aqueous phase and did not possess any stable conformation. In contrast, the main body of the protein is relatively compacted and conformationally rigid (Figure 3B). The simulation of LC3B-a reveals that its C-terminal conformation is far less mobile. It is bound to the main body of the protein in a helical form, as illustrated by simulation snapshots of the backbone conformation (Figure 3B).

The absence of Arg68 disrupts a salt bridge between the side chains of Arg70 and Asp48 which is also an important internal interaction in LC3 [41]. Also, the absence of Arg68 leads to a reduction of one rotation in the α3 helix, which is important in stabilizing the interaction between loops L1 (a.a. 38–50) and L2 (a.a. 71–79) (Figure 3C) and maintaining the ubiquitin fold structure. The absence of one rotation in the α3 helix takes the two loops apart, disrupting the hydrophobic cluster formed by Leu44, Pro45, Leu47, Leu71, Leu73, Phe79, and Tyr113 in LC3B, leaving a weaker interaction among only three residues, Leu44, Phe78 and Tyr112 in LC3B-a (Figure 3C). Together, these changes significantly weakens the dynamic interaction between loops L1 and L2, allowing the exposed hydrophobic groove between them to accommodate the hydrophobic side chain of Thr117, Phe118, Met120, Leu122 and Val124 in the C-terminus of LC3B-a. Thus, the C-terminal tail of LC3B-a may attach to the main protein body, shielding the hydrophobic chain from solvent and preventing the cleavage by the scissile peptide bond of human ATG4 family proteins, as shown by the space-filling models of the proteins (Figure 3D).

The MD simulation revealed that the intramolecular interaction in LC3B-a is weaker than that of LCB. To confirm this, we analysed their far-UV circular dichroism spectra under different temperatures. As shown in Figure 3E, the spectra of these two isoforms were similar at room temperature, indicating that the overall structure of LC3B-a did not change significantly from that of LC3B. However, as the temperature increased, LC3B-a lost its characteristic minimum ellipticity of the α helix at 208 nm at 45°C. In contrast, this signal of LC3B did not change until the temperature reached 65°C. These results suggest that LC3B-a is thermally less stable than LC3B.

In addition, we also used trypsin digestion of LC3B and LC3B-a to assess their conformational flexibility. As shown in Figure 3F, LC3B was cleaved into one main short fragment within the first 5 minutes and the second short fragment appeared after 30 minutes of trypsin digestion. In contrast, LC3B-a was quickly cleaved to at least five small fragments in the first 5 minutes. Thus, LC3B’s main body had a more compact and stable structure in solution than LC3B-a, despite its more flexible C-terminus. These results suggest that the intramolecular interactions of LC3B-a is weaker than that of LC3B and the C-terminus of LC3B-a may be accommodated into the exposed hydrophobic groove, which inhibits the cleavage by ATG4 and inhibits the whole post-translational modification process.

### Arg68 is essential for the interaction between human Atg8 family proteins and ATG4B

**Figure 4:**
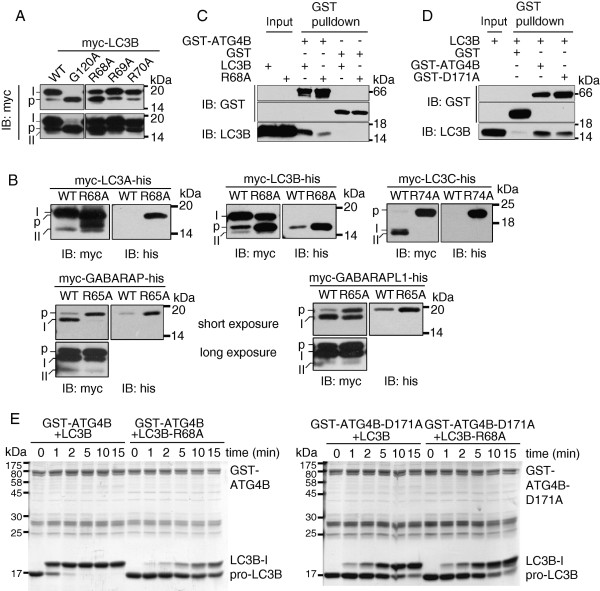
**Arg68 of LC3B is essential for protein-protein interaction with ATG4B and for C-terminal cleavage efficiency of human Atg8 family proteins. A**. Post-translation modification pattern of wild type (WT) and mutants of LC3B. Myc-LC3B-G120A mutant shows the position of pro-form protein. Long exposure (lower panel) shows the PE-conjugated forms. p: pro-form; I: the -I form; II: the -II form. **B**. The function of R68 of LC3B in post-translational modification is conserved among human Atg8 family members. Compared with wild type protein, R68A mutant of LC3A, R74A mutant of LC3C, R65A mutant of GABARAP or GABARAPL1 shows accumulated pro-form of each protein. Long exposure shows the -II form in GABARAP and GABARAPL1. **C**. Disruption of the salt bridge between R68 of LC3B and D171 of ATG4B weakens their interaction. GST pull-down assay was performed between GST-ATG4B with LC3B or LC3B -R68A protein in the presence of 1mM PMSF. **D**. GST pulldown assay was performed between LC3B with GST-ATG4B or GST-ATG4B-D171A mutant proteins in the presence of 1mM PMSF. **E**. Arg68-Asp171 salt bridge is crucial for the cleavage efficiency of LC3B by ATG4B. Purified GST-ATG4B, GST-ATG4B-D171A, LC3B and LC3B-a mutant proteins were subjected to *in vitro* cleavage assay in a time dependent manner. The cleavage pattern at indicated time point is shown by SDS-PAGE followed by coomassie bright blue staining. Molecular weights (kDa) were shown. IB: immunoblotting.

Three continuously conserved arginines (Arg68, Arg69, and Arg70) are located together in the α3 helix of LC3B. Thus, to determine whether these arginine residues are essential for their autophagic function, we mutated each of them to alanine and analysed their post-translational modification patterns by western blotting. To our surprise, as shown in Figure 4A, the R68A mutation had significantly accumulated pro-form of LC3B, while the modification patterns of R69A and R70A mutants were almost the same as the wild type protein (Figure 4A), suggesting that Arg68, not Arg69 or Arg70, is essential for the C-terminal cleavage of LC3B. This effect of Arg68 in LC3B is also conserved in other homologs of human Atg8 proteins. As shown in Figure 4B, mutation of the corresponding Arg68 of LC3B (Arg68 of LC3A, Arg74 of LC3C, Arg65 of GABARAP and GABARAPL1) to alanine also results in the accumulation of the pro-form of each protein, suggesting that the biological function of Arg68 is conserved among human Atg8 homologs.

The three continuously conserved arginines (Arg68, Arg69, and Arg70) are located together in the α3 helix of LC3B, which may form a local positively charged area and serve as a potential protein-protein interaction sites. As mentioned above, only Arg68 can affect the cleavage of human Atg8 family proteins. Thus, we wondered whether Arg68 was an essential residue for the interaction with human ATG4 family proteins. Since the crystal structure of the complex between rat LC3 and human ATG4B has been solved [42], we took advantage of these structures to examine the amino acids likely to be involved in the protein-protein interactions. As shown in Additional file 1: Figure S5, the side chain of Arg68 points to the interface of these two proteins, forming a salt bridge with Asp171 of ATG4B. We then compared the binding affinity of LC3B and LC3B-R68A with ATG4B in the presence of 1mM PMSF, which could inhibit the cysteine protease activity of ATG4B. As shown in Figure 4C and 4D, the interaction between these two proteins was decreased when mutating Arg68 of LC3B or Asp171 of ATG4B, indicating that the salt bridge between Arg68 and Asp171 is important for the LC3B-ATG4B interaction. We also performed a time-dependent *in vitro* cleavage assay using purified GST-ATG4B, GST-ATG4B-D171A, LC3B, and LC3B-R68A proteins. As shown in Figure 4E, wild type ATG4B and LC3B could finish their cleavage within 10 minutes, leaving only the up-shifted –I form protein. However, when the salt bridge was destroyed, there were still large amounts of pro-LC3B left in the reaction mixture after 20 minutes. Thus, the mutation of Arg68 or Asp171 of ATG4B impaired the cleavage efficiency significantly.

### Arg68 is essential for the autophagosome localization and autophagic flux

**Figure 5:**
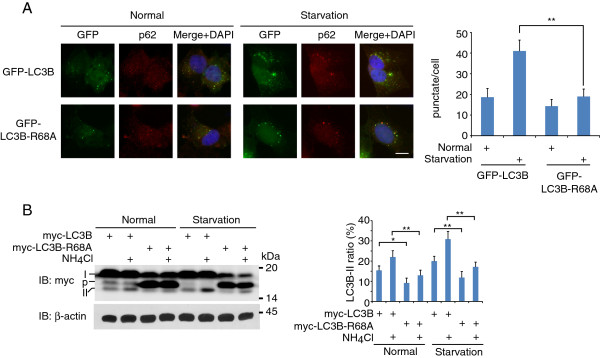
**Mutation of R68A impairs the autophagosomal localization and autophagic flux of LC3B protein. A**. Representative images of autophagosomal localization of GFP-LC3B and GFP-LC3B-R68A in normal culture condition and starvation conditions. GFP-LC3B and GFP-LC3B-R68A were stably expressed in HeLa cells. After treated with or without starvation for 2 hours, cells were subjected to immunostaining with p62 protein as autophagosome marker. The average punctate number in each cell is shown in the right. More than 100 cells were examined for the GFP puncta number and data were obtained from 3 independent experiments and represented as mean ± SEM. Bar = 20 μm. **B**. Autophagic flux was determined by monitoring LC3B-II turnover. HeLa cells expressing myc-LC3B or myc-LC3B-R68A were treated with or without 20mM NH_4_Cl for 4 hours in normal culture condition or starvation condition. Then the cell lysates were subjected to SDS-PAGE and immunobloting with anti-myc and anti-β-actin antibodies. The ratios of LC3B-II in the total LC3B protein according to the intensity of band were then plot on the right. Data were obtained from 3 independent experiments and represented as mean ± S.D. * p < 0.05, ** p < 0.01.

Arg68 mutation did not abolish the PE-conjugation (Figure 4B, long exposure), indicating that R68A mutant proteins can go through the post-translational modification. However, the functional importance of Arg68 in mammalian cells was still unknown. Thus, we stably expressed GFP-LC3B or GFP-LC3B-R68A in HeLa cells, and counted their autophagosomal localization under normal culture condition or starvation condition. As shown in Figure 5A, GFP-LC3B-R68A showed a significant decrease of autophagosome puncta number in both conditions. Furthermore, after the starvation stimulation, the increase of GFP-LC3B-R68A puncta was much lower than that of GFP-LC3B, indicating that the LC3B-R68A mutant protein has lower autophagic activity in mammalian cells.

We also measured the autophagic flux by monitoring the LC3B turnover. HeLa cells expressing either myc-LC3B or myc-LC3B-R68A were supplied ammonium chloride (NH_4_Cl), a lysosomotropic reagent which attenuates the acidification of the lysosome and inhibits the degradation of LC3B-II, resulting in the accumulation of LC3B-II. Thus, the difference of LC3B-II ratio (LC3B-II/ total LC3B) between the samples in the presence or absence of NH_4_Cl represents the amount of LC3B delivered and degraded in lysosomes. As shown in Figure 5B, the LC3B-II ratio increase of wild type LC3B is significantly higher than that of LC3B-R68A mutant protein in both normal and starvation conditions, indicating the autophagic flux of LC3B-R68A is lower than that of wild type LC3B.

Both the results of autophagosomal localization and autophagic flux suggest that the LC3B-R68A mutant has lower autophagic activity in mammalian cells. As mentioned above, Arg68 of LC3B or the equivalent arginines in other homologs are key amino acids for protein-protein interaction between human Atg8 homologs and ATG4B. The mutation of Arg68 may decrease the cleavage efficiency by ATG4B, thus affecting its function in the autophagy process.

## Discussion

The post-translational processing of LC3B play essential roles in autophagy, especially in the formation of autophagosomes. In our previous work, we identified three members of the human LC3 family, LC3A, LC3B, and LC3C [22]. In that paper, we showed that LC3B could not undergo post-translational modification as LC3A and LC3C. In contrast, Tanida *et al.* reported that the C-terminus of LC3B could be cleaved to expose the conserved Gly120 for further modification [35]. To verify our observations, we sequenced the plasmids used in our previous work and found that some of our *LC3B* plasmids used in the previous paper were *LC3B-a*, the new alternative splicing isoform of *LC3B* reported in this paper. This explained our different result with that reported by Tanida *et al.* about the post-translational modification of LC3B.

Alternative splicing is an important mechanism for controlling gene expression and creating the great proteomic complexity from a limited number of genes [43]. The NAGNAG alternative splicing which happens in the human *LC3B* gene is actually widespread in the human genome, occurring in 30% of human genes [32]. The NAGNAG motif is also conserved in *LC3B* and *LC3C* genes in different species, including Homo sapiens, Pan troglodytes, Canis lupus familiaris, Bos Taurus, as well as the *LC3B* gene of Rattus norvegicus and *LC3C* gene of Gallus gallus. Protein isoforms resulting from the NAGNAG motifs have also been shown to display functional diversity, such as signaling activity [44], cellular localization [45], DNA binding activity [46], protein-protein interaction affinity [47]. Here, we report that LC3B-a has distinct post-translational modification patterns with that of LC3B. Since LC3B-a can interact with ATGB but cannot be cleaved by ATG4B, LC3B-a may have inhibitory effect of human Atg8 family proteins cleavage by ATG4B; this needs further investigation. Atg8 and its homologs were found to be the only protein associating with all autophagic related structures [12,23]. Its functions were largely unknown for a long time until recently Nakatogawa *et al.* reported that Atg8 is responsible for the autophagic membrane tethering and hemifusion [20] and cargo receptor recognition [48]. The structures of Atg8 homologs are important in understanding their functional mechanisms. Amar *et al.* have identified two sites which are required for autophagy by mutational analyses [49,50]. Phe77 and Phe79 of Atg8, equivalents of Phe80 and Leu82 in LC3B are part of the Atg4 recognition site, and Tyr49 and Leu50 are important for the downstream lipidation steps. Through alternative splicing analysis, we have found Arg68 as a new protein interaction site with ATG4B, which is important for the initial post-translational modification process of human Atg8 family proteins.

## Conclusions

The alternative splicing pattern analysis of human Atg8 family proteins revealed that the Arg68 of LC3B and the corresponding arginine in other human Atg8 family members is an essential residue for the post-translational modification of these ubiquitin-like molecules. In addition to its role in stabilizing the ubiquitin-core structure, Arg68 also plays an important role in the interaction between Atg8 family proteins and ATG4B proteins.

## Methods

### Allele-specific semi-quantification PCR amplification and sequencing

Allele-specific PCR primers were designed according to the method of Simsek *et al.*[51] and listed in Additional file 1: Table S2. Human MTC Panel I (Cat. No. 636742) and Human MTC Panel II (Cat. No. 636743) (Clontech) were used as PCR templates and the semi-quantification PCR was performed according to the manufacturer’s instructions. PCR products were then resolved on 2% agarose-EB gels.

For DNA sequencing, we used ABI PRISM Big Dye Terminator Cycle Sequencing V2.0 Ready Reaction Kit and the ABI PRISM 3730 DNA analyzer (Applied Biosystems, Foster City, Calif., USA) according to the manufacturer’s instructions.

### Plasmids and antibodies

Genes were cloned to pCMV-myc, pGEX-4T-1, and pEGFP-c1 vectors according to the standard protocol. The mutants of each gene were generated by using the QuikChange site-directed mutagenesis kit (Stratagene). Primers for cloning and mutation are listed in Additional file 1: Table S3. Anti-myc, anti-his and anti-GST monoclonal antibodies were purchased from Upstate. Anti-p62 antibody was purchased from abcam (ab56416). Rabbit anti-LC3B antibody was raised against human LC3B full length protein.

### Cell culture, transfection and Western blot

HeLa cell was cultured in Dulbecco’s Modified Eagle Medium with 10% fetal bovine serum. For starvation induction, cells were washed three times with PBS and incubated with Earle’s balanced salt solution (ESBB; Invitrogen) at 37°C for indicated time. For NH_4_Cl inhibition, 20mM NH_4_Cl was added to medium for 4 hours.

Cells were transfected with lipofectamine (Invitrogen) according to the manufacturer’s instructions. 24 hours after transfection, cells were subjected to further analysis. Stable cell line preparation was performed following standard protocols.

### Protein expression, purification and pulldown assay

The GST fusion proteins were expressed in BL21(DE3) *E. coli.* cells and purified using a glutathione-Sepharose 4B column (Novagen) followed by cleavage of thrombin (Novagen) if needed. All purified proteins were dialysis against PBS prior to use or storage.

2 μg GST or GST-ATG4B protein were incubated with 5 μg LC3B or LC3B mutant proteins with Glutathione Sepharose 4B beads (GE Healthcare) at 4°C for 2 hours in the presence of 1mM PMSF with rotation. Then the beads were thoroughly washed in ice-cold PBS for 5 times and then boiled in the SDS sample buffer for further analysis.

### Immunofluorescence confocal microscopy and quantification of the GFP-LC3 puncta

Cells stably expressing indicated GFP fusion proteins were treated with normal or starvation conditions. Then they were washed with ice-cold PBS 3 times and fixed in 4% paraformaldehyde at room temperature for 5 minutes. Then the cells were treated with 0.5% Triton X-100 in PBS at room temperature for 5 minutes. Cell samples were then blocked with 8% goat serum in PBS for 30 minutes and incubated with the primary antibody at room temperature for one hour. After three washing with PBS, samples were then incubated with the second antibody at room temperature for 30 minutes and then co-stained with DAPI for one minute. After 3 times of final washing by PBS, cells were then mounted and observed by using a 63x Oil immersion objective using a ZEISS LSM 710 Laser Scanning Confocal Microscope. The quantification of GFP puncta was performed by Image J version 1.38 (National Institutes of Health). More than 100 cells were quantified in each sample.

### Constructions of initial structures of LC3B and LC3B-a for molecular simulations

The crystal structure of rat LC3 (Protein Data Bank code: 1UGM) [38] was employed as the template for homology modeling to construct the initial structure of LC3B, as the amino acid sequence of rat LC3B is almost identical to that of LC3B. The initial structure of LC3B was built by directly submitting the sequence and structural template to the Swiss-MODEL server (http://swissmodel.expasy.org) [52]. This homology modeling based on 1UGM gave only the initial atomic coordinates of amino acids 5–117, because 1UGM lacks the coordinates of other amino acids. Since this study was intended to investigate the entire C-terminal region of the protein, we constructed the initial coordinates for amino acids 3–4 and 118–120 according to the crystal structure of another homologous protein GATE-16 by directly employing the backbone (ϕ, φ) angles of the corresponding amino acids (Protein Data Bank code: 1EQ6)[41]. Finally, the initial coordinates of remaining amino acids were determined by randomly setting their backbone rotational angles (ϕ,ψ) without any atomic overlap. With the initial structure, we performed MD simulations for LC3B as described below. Then, we used the simulation snapshot structure of LC3B at 1 ns to construct the initial structure of LC3B-a via the Swiss-MODEL server.

### MD simulations with explicit solvent representation

MD simulations were performed with the all-hydrogen force fields OPLS-AA [53,54] using the program suite GROMACS 3.1.4. [55,56]. The TIP4P water model48 was employed. To construct a simulation system, initial all-hydrogen structure of LC3B (or LC3B-a) was merged into a rectangular box of water, in which the water molecules were placed randomly without atomic overlap. The thickness of the water layer between the protein and the closest box-boundary is ~1.4 nm. As such, the simulation system for an isoform contains ~9,700 water molecules for the simulated water density of ~1 g/cm^3^. Finally, counterpart ions were placed into the box to make the system neutral. For all the simulations, we used the ensemble of constant number of molecules, pressure, and temperature (N-P-T ensemble), with the temperature held at 300 K and the pressure at 1 atm. In the simulations, the Berendsen temperature coupling method [57] was used with a coupling constant of 0.1 ps. Cutoff distance for van der Waals force was set to 1.0 nm. Electrostatic forces were calculated by the particle mesh Ewald (PME) method50 with a cutoff of 1.0 nm for real-space interactions, and the reciprocal-space interactions were computed on a 0.12 nm grid with fourth-order spline interpolation. The simulations were run under periodical boundary conditions using a time step of 2 fs. Two parallel simulations were performed for LC3B-α and LC3B-β. The period for each simulation run is 13 ns. Such a simulation time-scale is usually needed for the C-terminal segment which initial coordinates of 5 amino acids were randomly assigned to adjust to its conformations to the equilibrium states.

### Circular dichroism spectroscopy

Circular dichroism (CD) spectra were recorded at pH 7.0 in a Jasco J-810 spectropolarimeter equipped with a Peltier temperature control unit. The concentrations of proteins were 40 μM for the far-UV measurements (185–260 nm, light path 1 mm). The spectra obtained were the average of 4 scans. The absorbance from the buffer control was subtracted from the protein spectra. Thermal transition curves (25°C to 75°C) were determined by monitoring the decrease in ellipticity at 222 nm at a scanning rate of 40°C/h using a 1 mm path-length cell.

### Trypsin digestion

An aliquot of 50 ng trypsin was added to 20 μL PBS (pH = 7.4) containing 1 μg LC3B or LC3B-a, followed by incubation in 37°C water bath. Aliquots of the reaction mixture were taken out at the indicated time interval, quenched with 5 μL 5X gel loading buffer and heated at the 98°C water bath for 8 min. The samples were resolved on 4% ~ 12% NuPAGE^®^ Novex Bis-Tris gels that were stained with silver.

### In vitro ATG4B cleavage assay

6 μg of GST-ATG4B or GST-ATG4B-D171A proteins were mixed with 12 μg LC3B or LC3B-R68A proteins in 120 μl reaction volume of PBS, followed by incubation in 30°C water bath. At the time of 0, 1, 2, 5, 10 minutes, 20 μl reaction mixtures were subjected to sampling preparation. All the samples are subjected to SDS-PAGE and Commassie Blue Staining.

### Autophagic flux determination

1 μg construct of myc-LC3B or myc-LC3B-R68A was transfected into 6-well plate of HeLa cells. 24 hours after transfection, cells were treated with normal or starvation conditions in the presence or absence of 20mM NH4Cl for 4 hours. Cell lysates were then subjected to SDS-PAGE and immunobloting with anti-myc antibody. Fluorescence signals were obtained by Bio-Rad ChemiDoc™ XRS + System and bands signals were quantified by Image Lab™ Software version 2.0.

## Abbreviations

Phosphatidylethanolamine: PE; Expressed sequence tags: EST; Molecular dynamics: MD.

## Competing interests

The authors declare that they have no competing interests.

## Authors’ contributions

CL, HM, and JW performed most of the experiments. QH performed the MD simulation. JOL, LY designed the experiments and analysed data. CL wrote the paper. All authors read and approved the final manuscript.

## Supplementary Material

Additional file 1: Table S1 Gene Bank accession numbers of ESTs. **Table S2.** Allele specific primers for each transcript. **Table S3.** Primers used in this paper for cloning and mutagenesis. **Figure S1.** The sequencing results of *LC3B-a*, *GABARAP-a* and *GABARAPL1-a.***Figure S2.** Primer specificity test for LC3B and LC3B-a transcripts. **Figure S3.** Alignment of human Atg8 family proteins. **Figure S4.** The interaction between LC3B-a and ATG4B is intact as that of LC3B. **Figure S5.** Cartoon representation of human ATG4B-rat LC3B complex. Click here for file
