# Supplementary material for: Arginine68 is an essential residue for the C-terminal cleavage of human Atg8 family proteins
Source: BMC Cell Biol. 2013 May 30;14:27. doi: 10.1186/1471-2121-14-27 (PMC3686597; doi:10.1186/1471-2121-14-27)

Supplemental Information

Supplemental Table S1 Gene Bank accession numbers of ESTs

By searching the ESTs database, we listed the representative ESTs which could support the existence of each transcript.

Supplemental Table S2 Allele specific primers for each transcript

Supplemental Table S3 Primers used in this paper for cloning and mutagenesis

Supplemental Figure S1 The sequencing results of *LC3B-a*, *GABARAP-a* and *GABARAPL1-a*

To verify the potential alternative spliced transcript, products of RT-PCR were ligated into T-vector. Then these T-vectors were subjected to DNA sequencing. The starting codon and stop codon were annotated with boxes. The alternative splicing positions were annotated with arrow heads.

Supplemental Figure S2 Primer specificity test for LC3B and LC3B-a transcripts

LC3B-sense primer was used together with LC3B-antisense primer or LC3B-a-antisense primer to amplify 10pg of myc-LC3B or myc-LC3B-a plasmid. The amplification conditions were as follows: 95ºC for 3 min; 35 cycles of 95 ºC for 10 s and 65 ºC for 15 s; final extension at 65 ºC for 1min. Then PCR results were analysed by agarose electrophoresis.

Supplemental Figure S3 Alignment of human Atg8 family proteins

Protein sequences of human Atg8 family members and Atg8 from *Saccharomyces cerevisiae* are aligned. The conserved Glycine in the C-terminus is indicated by arrow and the Arg68 lost in LC3B-a is indicated by arrow head. All the residues are numbered after LC3B. Secondary structures base on Atg8 are shown under the sequences.

Supplemental Figure S4 The interaction between LC3B-a and ATG4B is intact as that of LC3B. The interactions between ATG4B with LC3B or LC3B-a were compared by GST pulldown assay. 2 μg GST-ATG4B and 5 μg LC3B or LC3B-a were subjected to GST pulldown assay in the presence of 1mM PMSF. The proteins binding with beads were then resolved by SDS-PAGE and western blot with anti-GST and anti-LC3B antibodies. Molecular weight is shown on the right.

Supplemental Figure S5 Cartoon representation of human ATG4B-rat LC3B complex

Human ATG4B-rat LC3B complex (2Z0D) showed the salt bridge between R68 and D171 (yellow dashes in the enlarged box).

Supplemental Table S1

Transcript Name	GenBank Accession No.
LC3B	BE789120 BI604136 BU599096 DA733952 DA830923 W60415 etc.
LC3B-a	DB110930 DA838047 DA298782 CN481672 DB110684 DA378385 DA145209 BQ228361 DB128770 DA464826 DA059449 BI666532 DB130435 DA575770 DA169122 BG708318 DB115486 DA575751 DA055097 DA779844 DA713396 DA653807 DA085702 DA697312 DA838441 DA155225 BP243235 BP234072
GABARAP	AB030711 AF044671 AF067171 AF161586 AF183425 AK098634 etc.
GABARAP-a	DA613815 etc.
GABARAPL1	AA476809 AF087847 AF287012 AK074195 AL136676 AL529653 etc.
GABARAPL1-a	AL702909 BP228611 etc.

Supplemental Table S2

Primer Name	Sequence (5' to 3')
LC3B-sense	ATGCCGTCGGAGAAGACCTTCAA
LC3B-antisense	TTAGCATTGAGCTGTAAGCGCCTTC
LC3B-a-antisense	GCATTGAGCTGTAAGCGCCTAA
GABARAP-sense	GGAGGATGAAGTTCGTGTACAAAGAAGAG
GABARAP-antisense	CAGCAGCTTCACAGACCGTAGACAC
GABARAP-a-antisense	CAAAGCCTCCACCACTTCCCAG
GABARAPL1-sense	TCTCCATCTGGCTCTCCTCTACCTCC
GABARAPL1-antisense	GGCTTCCAACCACTCATTTCCCATA
GABARAPL1-a-antisense	CAAGAGGTATCTAATCTCTCAGAGCCTTACAC
B2M-sense	ATGAGTATGCCTGCCGTGTGAAC
B2M-antisense	TGTGGAGCAACCTGCTCAGATAC

Supplemental Table S3

Primer Names	Primer Sequence (5' to 3')	Note
myc-A-sense	CCGAATTCGGATGCCCTCAG	cloning LC3A into pCMV-myc vector
A-his-antisense	AAGGTACCTCGAGTTAATGGTGATGGTGATGATGGAAGCCGAAG	adding his-tag to the C-terminal of LC3A and cloning LC3A into pCMV-myc vector
myc-C-sense	CCGAATTCGGATGCCGCCTC	cloning LC3C into pCMV-myc vector
C-his-antisense	GCGGTACCTCGAGTTAATGGTGATGGTGATGATGGAGAGGATTG	adding his-tag to the C-terminal of LC3C and cloning LC3C into pCMV-myc vector
myc-B-sense	CCGAATTCGGATGCCGTCGG	cloning LC3B or LC3B-a to pCMV-myc vector
B-his-antisense	AAGGTACCTTAATGGTGATGGTGATGATGCACTGACAAT	adding his-tag to the C-terminal of LC3B or LC3B-a and cloning into pCMV-myc vector
myc-G-sense	CGAGATCTCAATGAAGTTCG	cloning GABARAP or GABARAP-a to pCMV-myc vector
G-his-antisense	AAGGTACCTCGAGTTAATGGTGATGGTGATGATGCAGACCGTAG	adding his-tag to the C-terminal of GABARAP and cloning into pCMV-myc vector
myc-L1-sense	CCGAATTCGGATGAAGTTCC	cloning GABARAPL1 into pCMV-myc vector
L1-his-antisense	AAGGTACCTTAATGGTGATGGTGATGATGTTTCCCATAG	adding his-tag to the C-terminal of GABARAPL1 and cloning into pCMV-myc vector
G-a-his-antisense	AAGGTACCTCGAGTTAATGGTGATGGTGATGATGTCACCATACCTG	adding his-tag to the C-terminal of GABARAP-a and cloning into pCMV-myc vector
L1-a-his-antisense	AAGGTACCTTAATGGTGATGGTGATGATGTCAGAGCCTTA	adding his-tag to the C-terminal of GABARAPL1-a and cloning into pCMV-myc vector
B-antisense	GCGGTACCTTACACTGACAAT	cloning LC3B or LC3B-a into pCMV-myc vector
GST-4B-sense	GTGGATCCATGGACGCA	cloning ATG4B into pGEX-4T-1 vector
GST-4B-antisense	AACTCGAGTCAAAGGGACAG	cloning ATG4B into pGEX-4T-1 vector
GST-B-sense	CGTGGATCCATGCCGTCGG	cloning LC3B or LC3B-a into pGEX-4T-1 vector
GST-B-antisense	CGCTCGAGCACTGACAAT	cloning LC3B or LC3B-a into pGEX-4T-1 vector
GFP-B-sense	TCGAATTCGATGCCGTCGG	cloning LC3B or LC3B-a into pEGFP-c1 vector
GFP-B-antisense	GCGGTACCTTACACTGACAATTTC	cloning LC3B or LC3B-a into pEGFP-c1 vector
GFP-G-sense	TCAGATCTATGAAGTTCG	cloning GABARAP into pEGFP-c1 vector
GFP-G-antisense	GCGGTACCTTACAGACCGTAG	cloning GABARAP into pEGFP-c1 vector
GFP-G-a-sense	ATCTCGAGCTATGAAGTTCGT	cloning GABARAP-a into pEGFP-c1 vector
GFP-G-a-antisense	GCGGTACCTCACCATACCTGGT	cloning GABARAP-a into pEGFP-c1 vector
GFP-L1-sense	TCGAATTCGATGAAGTTCC	cloning GABARAPL1 into pEGFP-c1 vector
GFP-L1-antisense	GCGGTACCTTATTTCCCATAG	cloning GABARAPL1 into pEGFP-c1 vector
GFP-L1-a-sense	ATCTCGAGCTATGAAGTTCC	cloning GABARAPL1-a into pEGFP-c1 vector
GFP-L1-a-antisense	GCGGTACCTCAGAGCCTTAC	cloning GABARAPL1-a into pEGFP-c1 vector
B-R68A-sense	GTGAGCTCATCAAGATAATTGCGAGGCGCTTACAGCTCAATGC	mutating R68A of LC3B
B-R68A-antisense	GCATTGAGCTGTAAGCGCCTCGCAATTATCTTGATGAGCTCAC	mutating R68A of LC3B
B-R69A-sense	GCTCATCAAGATAATTAGAGCGCGCTTACAGCTCAATGC	mutating R69A of LC3B
B-R69A-antisense	GCATTGAGCTGTAAGCGCGCTCTAATTATCTTGATGAGC	mutating R69A of LC3B
B-R70A-sense	TCATCAAGATAATTAGAAGGGCGTTACAGCTCAATGCTAATCAG	mutating R70A of LC3B
B-R70A-antisense	TGATTAGCATTGAGCTGTAACGCCCTTCTAATTATCTTGATGAG	mutating R70A of LC3B
A-R68A-sense	GTTGGTCAAGATCATCGCGCGCCGCCTGCAGCTG	mutating R68A of LC3A
A-R68A-antisense	CAGCTGCAGGCGGCGCGCGATGATCTTGACCAAC	mutating R68A of LC3A
C-R74A-sense	CCAGTTCCTCAGCATCATCGCCAGCCGCATGGTCCTGAGAG	mutating R74A of LC3A
C-R74A-antisense	CTCTCAGGACCATGCGGCTGGCGATGATGCTGAGGAACTGG	mutating R74A of LC3A
G-R65A-sense	GTCAGTTCTACTTCTTGATCGCGAAGCGAATTCATCTCCGAG	mutating R65A of GABARAP
G-R65A-antisense	CTCGGAGATGAATTCGCTTCGCGATCAAGAAGTAGAACTGAC	mutating R65A of GABARAP
L1-R65A-sense	GCCAGTTCTACTTCTTAATCGCGAAGAGAATCCACCTGAGAC	mutating R65A of GABARAPL1
L1-R65A-antisense	GTCTCAGGTGGATTCTCTTCGCGATTAAGAAGTAGAACTGGC	mutating R65A of GABARAPL1
ATG4B-D171A-sense	CCACATTGCAATGGCCAACACTGTTGTGA	mutating D171A of ATG4B
ATG4B-D171A-antisense	TCACAACAGTGTTGGCCATTGCAATGTGG	mutating D171A of ATG4B
ATG4B-C74A-sense	AGGCTGGGGCGCCATGCTGCGGT	mutating C74A of ATG4B
ATG4B-C74A-antisense	ACCGCAGCATGGCGCCCCAGCCT	mutating C74A of ATG4B
B-G120A-antisense	GCGGTACCTTACACTGACAATTTCATCGCGAACGTCTC	mutating G120A of LC3B

Supplemental Figure S1

LC3B-a:

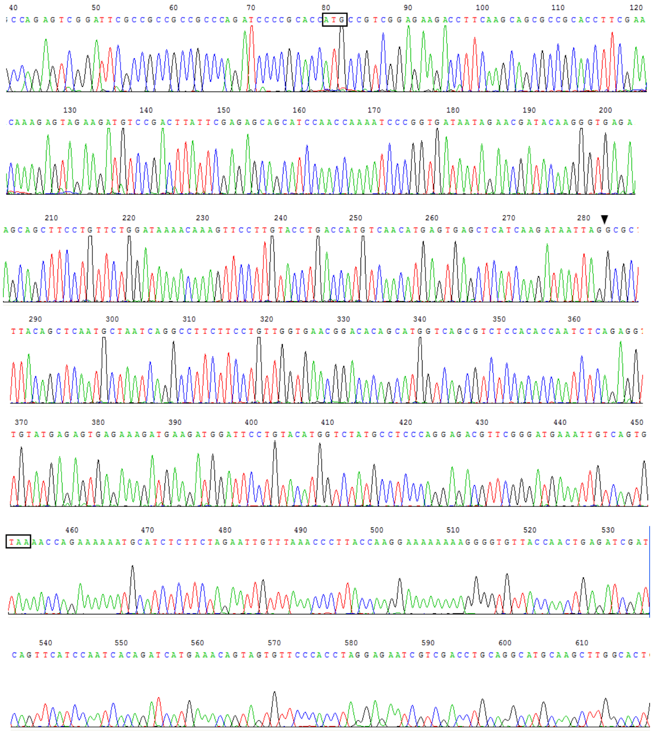

GABARAPL1-a:

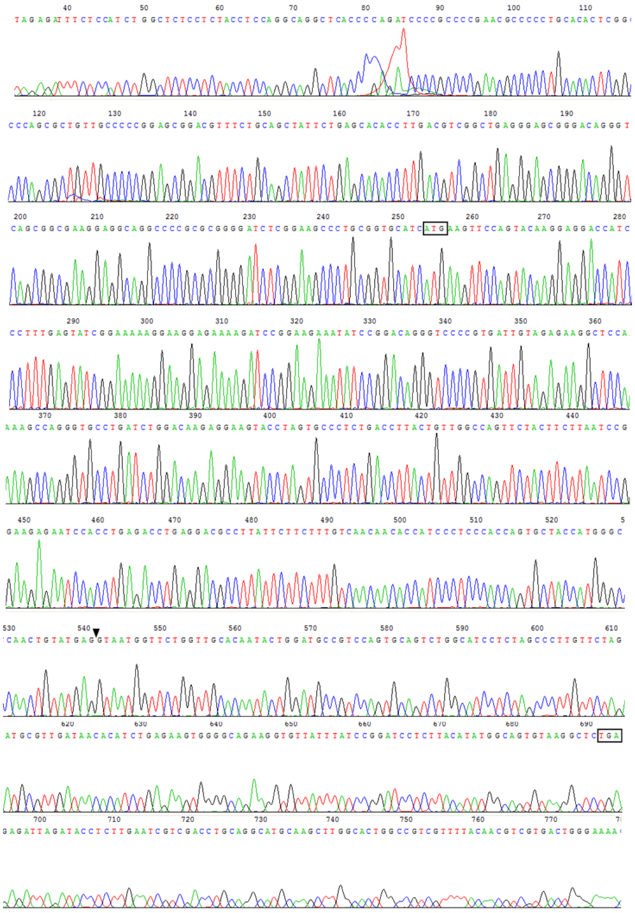

GABARAP-a:

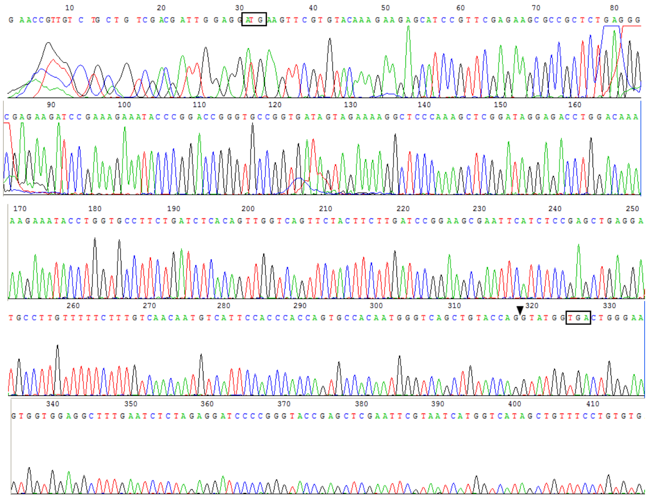

Supplemental Figure S2

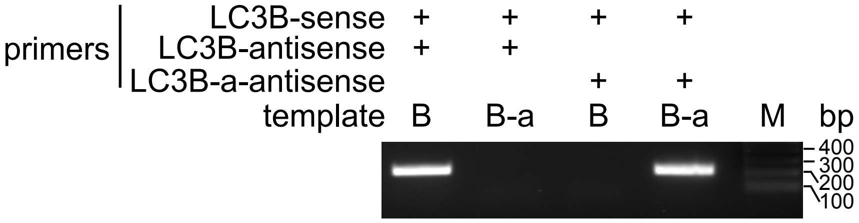

Supplemental Figure S3

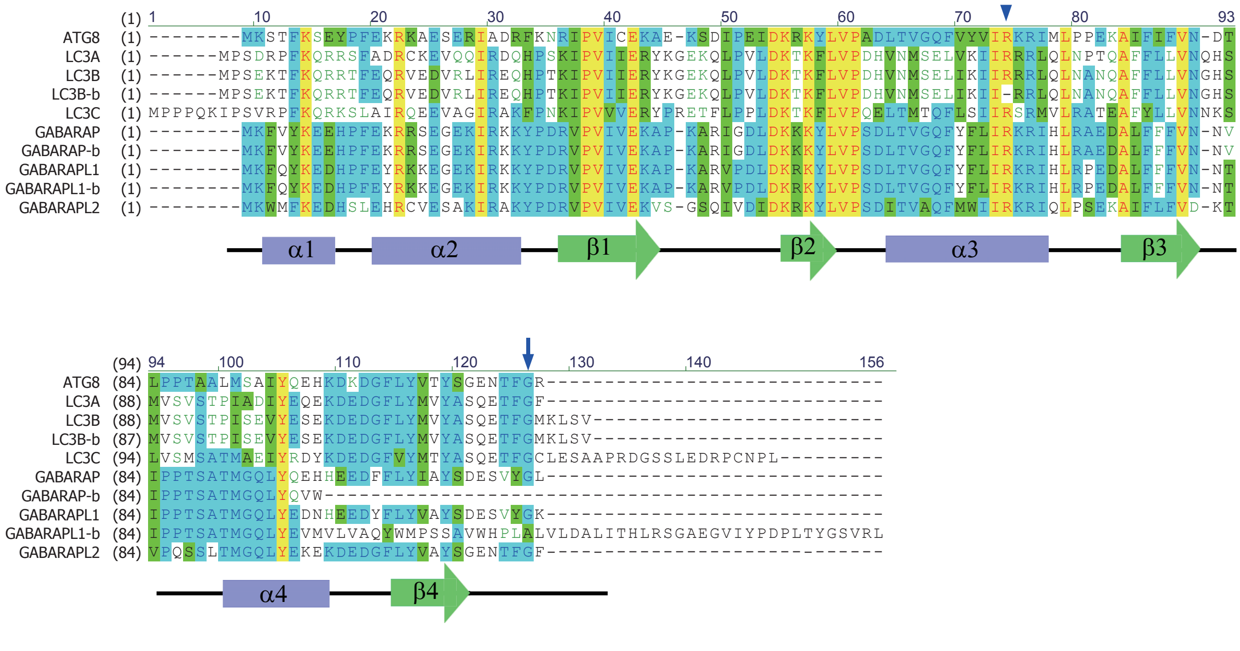

Supplemental Figure S4

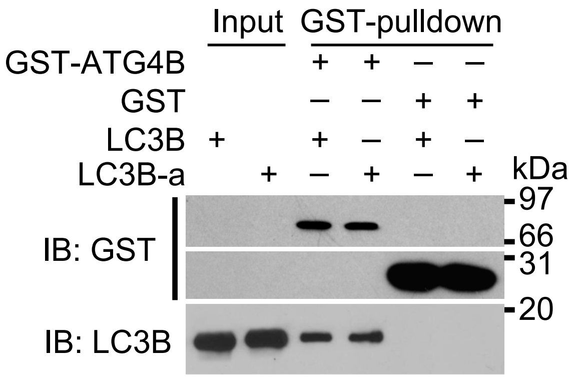

Supplemental Figure S5

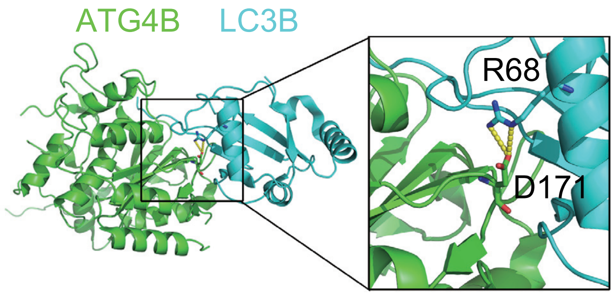

Supplement: Additional file 1: Table S1 — Gene Bank accession numbers of ESTs. Table S2. Allele specific primers for each transcript. Table S3. Primers used in this paper for cloning and mutagenesis. Figure S1. The sequencing results of LC3B-a, GABARAP-a and GABARAPL1-a.Figure S2. Primer specificity test for LC3B and LC3B-a transcripts. Figure S3. Alignment of human Atg8 family proteins. Figure S4. The interaction between LC3B-a and ATG4B is intact as that of LC3B. Figure S5. Cartoon representation of human ATG4B-rat LC3B complex. [file 1471-2121-14-27-S1.pdf]
